# Clinical Comparisons of Two Free Light Chain Assays to Immunofixation Electrophoresis for Detecting Monoclonal Gammopathy

**DOI:** 10.1155/2014/647238

**Published:** 2014-05-27

**Authors:** Han-Sung Kim, Hyun Soo Kim, Kyu-Sung Shin, Wonkeun Song, Hyo Jung Kim, Hyeong Su Kim, Min-Jeong Park

**Affiliations:** ^1^Department of Laboratory Medicine, Hallym University College of Medicine, Seoul 150-950, Republic of Korea; ^2^Department of Internal Medicine, Hallym University College of Medicine, Seoul 150-950, Republic of Korea

## Abstract

Free light chains (FLCs) are useful biomarkers for the diagnosis and monitoring of various plasma cell dyscrasias. One hundred fifty-seven samples from 120 patients for screening or monitoring of monoclonal gammopathy (MG) were included. The new N Latex FLC assays (Siemens Healthcare Diagnostics GmbH, Germany) were compared with the Freelite FLC assays (The Binding Site Ltd., UK) and the results were analyzed with those of immunofixation electrophoresis (IFE). The Freelite FLC assay showed significantly wider assay ranges than the N Latex FLC assay. The correlation coefficients of the two FLC kappa (**κ**) assays, lambda (**λ**) assays, and the **κ**/**λ** ratio were 0.9792, 0.8264, and 0.9064, respectively. The concordance rate was 84.7% for the FLC **κ** assays, 79.6% for FLC **λ**, and 89.2% for the **κ**/**λ** ratio. The clinical sensitivity and specificity of the **κ**/**λ** ratios were 72.2% and 93.6% for the Freelite assay and 64.6% and 100% for the N Latex FLC assay. Two FLC assays showed good correlations and concordance. However, the clinical sensitivity of the **κ**/**λ** ratio was higher in the Freelite FLC assays; clinical specificity was higher in the N Latex FLC assay. Both FLC assays seem to have limited clinical utility in detecting MG in certain clinical settings.

## 1. Introduction

Monoclonal gammopathy (MG) pertains to a disease cluster involving plasma cells: it is generally detected in the serum and urine and is characterized by a clonal synthesis of monoclonal proteins. To diagnose MG, test methods for detecting monoclonal proteins, such as electrophoresis, have been employed for decades. It has been recently shown that MG induces excessive production of immunoglobulin free light chains, which remain in the blood without being bound to heavy chains [1, 2]. A test method for measuring kappa (*κ*) and lambda (*λ*) free light chains (FLCs) and calculations for the kappa-to-lambda ratio have recently been introduced [3]. The test method, known as the Freelite assay (The Binding Site Group Ltd., Birmingham, UK), has been extensively used worldwide. FLCs are one of the response criteria used in the diagnosis and treatment of the myeloma and related disorders, as described by the International Myeloma Working Group guidelines [4]. However, despite the usefulness of this method, several analytical problems have continuously been faced, including lot-to-lot variability of reagents, antigen excess, unrecognizable epitopes, and excessive polymerization [5–8]. The Freelite assay has the limitations of poor postdilution linearity and relative imprecision, as well as increased likelihood of showing false negative results due to antigen excess in patients with extremely high FLC concentration [3, 7]. To overcome these problems, a new N Latex assay (Siemens Healthcare Diagnostics GmbH, Marburg, Germany) using monoclonal antibodies has been developed and recently made available [9].

This study aimed to analyze the performance of the newly developed N Latex FLC assay compared with the Freelite FLC assay in patients with various diseases and evaluate the clinical usefulness of the two FLC assays compared to the standard diagnostic test of immunofixation electrophoresis (IFE).

## 2. Materials and Methods

### 2.1. Patient Samples

During a 4-month period in 2012, from April to July, 157 pairs of samples of serum and urine were collected from 120 patients who had registered for laboratory screening or monitoring of MG. The study population consisted of 63 patients with MG (MG group) and 57 patients without MG (non-MG group). The clinical diagnoses of the patients in each group were determined by their physicians. The specimens in the MG group included those from newly diagnosed patients and patients undergoing treatment. The patients in the MG group had one or more monoclonal proteins in their serum or urine specimens detected on the IFE. The results of FLC assays were evaluated in relation to serum and urine IFE. This study was approved by the Institutional Review Board of Kangnam Sacred Heart Hospital in Republic of Korea.

### 2.2. FLC Assays

Two FLC assays for FLC *κ* and *λ* in serum were used: N Latex FLC assays (Siemens Healthcare Diagnostics GmbH, Marburg, Germany) using monoclonal Ab-based method and Freelite assays (The Binding Site Ltd., Birmingham, UK) using polyclonal Ab-based method. Both assays were performed on Behring Nephelometer II (Siemens Healthcare Diagnostics GmbH, Marburg, Germany). The N Latex assay was subjected to a prereaction step for 2 minutes prior to the assay, which allowed using a step with a higher dilution factor in the presence of a large amount of antigens, thereby reducing the false negatives caused by the antigen excess. In this study, both assays were performed by the next higher dilution factor, in addition to the automatically determined dilution factor, to preclude the possibility of false negatives caused by antigen excess.

The reference ranges of both assays were provided by the manufacturers and were as follows: N Latex FLC *κ*, 6.7–22.4 mg/L; Freelite *κ*, 3.3–19.4 mg/L; N Latex FLC *λ*, 8.3–27 mg/L; Freelite *λ*, 5.7–26.3 mg/L; N Latex FLC *κ*/*λ* ratio, 0.31–1.56; Freelite *κ*/*λ* ratio, 0.23–1.65.

### 2.3. Immunofixation Electrophoresis

The detection limit of IFE in serum is around 15 mg/dL. A total of 157 pairs of serum and urine samples were analyzed on IFE using an agarose gel (Helena Laboratories, Beaumont, USA). Any M-band detected in serum and/or urine IFE was considered positive for MG. The gels were evaluated by two blinded independent readers.

### 2.4. Comparison of Methods

We compared the principles behind the N Latex FLC assays and the Freelite assays. Correlation analysis was performed using the results for the *κ*, *λ*, and *κ*/*λ* ratios of the N Latex FLC and the Freelite assays. A qualitative comparison was performed by determining the concordance rate wherein both assays generated the same results (abnormal low, normal, and abnormal high). In addition, we investigated the cases showing discordant results between the two FLC assays.

### 2.5. Clinical Sensitivity and Specificity

The clinical sensitivity (true positive), specificity (true negative), and percent agreement of the *κ*/*λ* ratios of the N Latex FLC and Freelite FLC assays were calculated based on the clinical diagnosis and the results of the IFE analysis.

### 2.6. Statistical Analyses

Statistical analyses were performed using the STATA software (STATA SE v12.0, Stata Corp LP, Lakeway, USA). Spearman's rank correlation coefficients and Cohen's kappa for concordance analysis were performed. Normalized median differences pertain to the median value of all differences between the sample outcomes of the two methods as calculated in terms of percentage by [(*y* − *x*)/(*y* + *x*)]/2 [9].

## 3. Results

### 3.1. Clinical Characteristics of Study Populations

The study populations of 120 patients were shown in Table 1. The male to female ratio was 1.3 : 1 in the MG group and 1 : 1 in the non-MG group. No significant differences in age distribution were observed; the mean ± SD was 66.5 ± 9.8 in the MG group versus 69.3 ± 14.4 in the non-MG group (*P* > 0.05).

### 3.2. Comparisons of FLC Assays

#### 3.2.1. Quantitative Analysis

**Figure 1:**
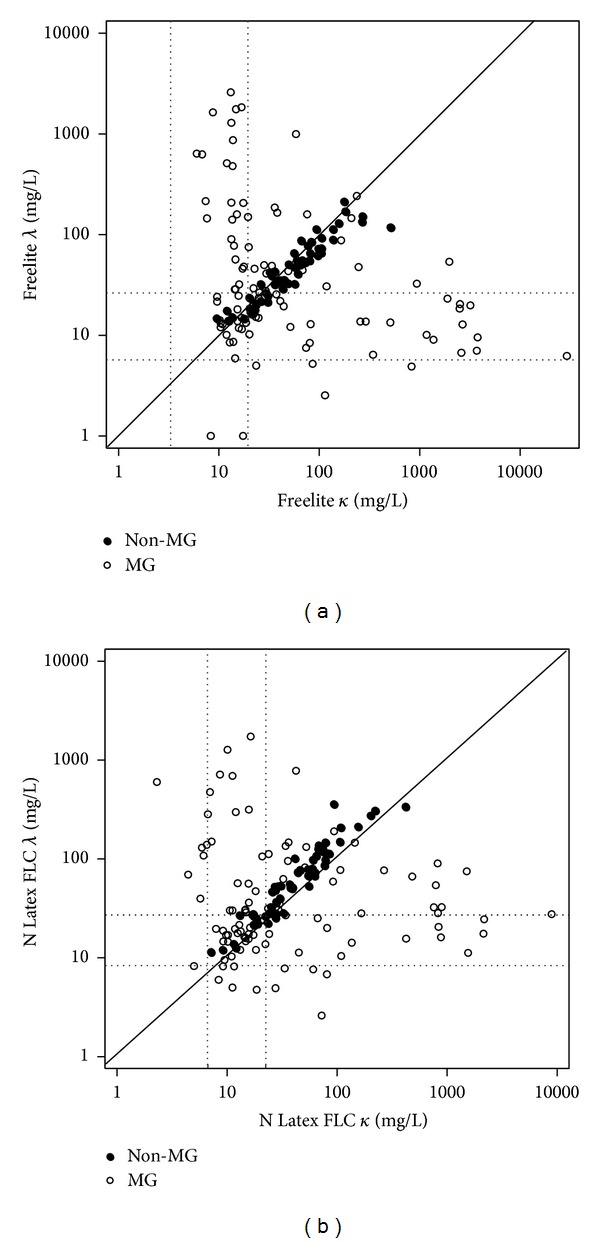
Free light chain *κ* versus *λ* for Freelite assay (a) and N Latex FLC assay (b) in 157 samples. Dotted lines indicate the reference ranges for the specific assays. The solid line indicates the *y* = *x* axes.

The results of the kappa and lambda FLC analyses, as measured by the two assays, for all the specimens, were plotted. The MG group showed a tendency towards the kappa or lambda axis depending upon the monoclonal type, whereas the non-MG group showed dense distribution around the line of identity in both assays. The Freelite assays revealed wider distributions of both *κ* and *λ* than the Latex assay (Figure 1).

**Figure 2:**
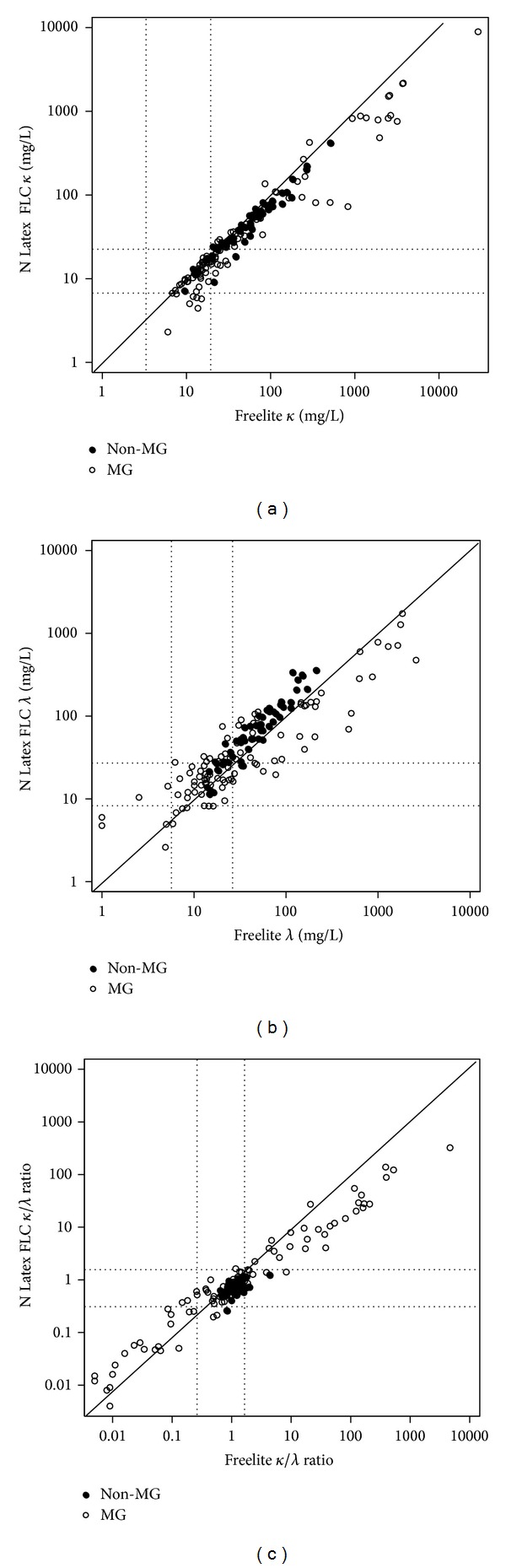
Method comparisons between Freelite assays and N Latex FLC assays for free light chain *κ* (a), *λ* (b), and *κ*/*λ* ratio (c) for 157 samples. Dotted lines indicate the reference ranges for the specific assays. The solid line indicates the *y* = *x* axes. MG: monoclonal gammopathy.

The kappa and lambda FLC measurements provided kappa values distributed in the ranges of 6.0–29,200.0 mg/L for the Freelite assay and 2.3–8,920.0 mg/L for the N Latex assay, respectively, indicating that the Freelite assay has at least a 3-fold broader range than the Latex assay, with an excellent Pearson's coefficient of 0.9792 and with a normalized difference of −4.6%. In the case of lambda, the ranges measured using the Freelite assay and the N Latex assay were 1.0–2,590.0 mg/L and 2.6–599.0 mg/L, respectively, demonstrating that the Freelite has at least a 4-fold broader range compared to the Latex, with a Pearson's coefficient of 0.8264, which denotes a relatively low correlation compared to kappa. In contrast to *κ*, the normalized difference of *λ* was 5.3%, indicating 5.3% lower median value in the Freelite assay. The maximum value of the *κ*/*λ* ratio of the Freelite assay was 4,687.0, which was at least 10 times higher than that of the Latex assay, 323.2. The normalized difference of *κ*/*λ* ratio was −10.1%, indicating 10% higher median value in the Freelite assay. Correlation coefficients between the *κ*/*λ* ratios were remarkably low in the non-MG group, 0.5168, compared to the MG group, 0.9065 (Table 2, Figure 2).

#### 3.2.2. Qualitative Analysis (Concordance Analysis)

**Figure 3:**
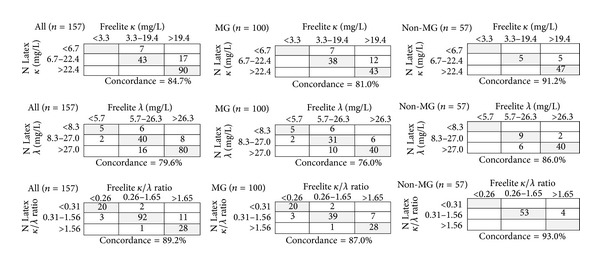
Concordance analysis for free light chain *κ*, *λ*, and *κ*/*λ* ratio of the Freelite assays and the N Latex FLC assays. MG: monoclonal gammopathy.

Based on the reference ranges provided by the manufacturers of both FLC assays, FLC results of the study population were classified into 3 groups, namely, abnormal high, normal, and abnormal low, and concordance rates were analyzed. The concordance rate between two measurements with respect to *κ*, *λ*, and *κ*/*λ* ratio was 84.7%, 79.6%, and 89.2%, respectively, for all the patients, whereas these were 81%, 76%, and 87%, respectively, in the MG group, and 91.2%, 86%, and 93%, respectively, in the non-MG group. The non-MG group exhibited a higher concordance rate for all the three items than the MG group. Cohen's kappa values of *κ*, *λ*, and *κ*/*λ* ratio were 0.70, 0.66, and 0.82, respectively. The value of *κ*/*λ* ratio, greater than 0.8, indicates a good concordance (Figure 3).

A total of 17 cases, 13 in the MG group and 4 in the non-MG group, showed discordance for the *κ*/*λ* ratio, as calculated from the two assays. Of the 13 cases in the MG group, the IFE results were in agreement with those of the Freelite assay results in 8 cases and with the Latex results in 5 cases. The 4 discordant cases in the non-MG group pertained to specimens from patients with chronic kidney disease (CKD), chronic obstructive pulmonary disease, iron deficiency anemia, and systemic lupus erythematosus (SLE), and all of them showed abnormal *κ*/*λ* ratios with the Freelite assay, suggesting false positive results compared to IFE. Taken together, the 17 discordant cases consisted of 8 (47.1%) Freelite and 9 (52.9%) N Latex results in agreement with the IFE results. Moreover, in the lambda FLC assay, 10 cases that showed normal results in the Freelite assay revealed increased values in the N Latex assay. Eight of these 10 cases were present in high concentrations of kappa-type M-proteins.

#### 3.2.3. Clinical Sensitivity and Specificity of the *κ*/*λ* Ratio of FLC Assays

The clinical sensitivity (true positive) and specificity (true negative) of the *κ*/*λ* ratio of N Latex FLC and Freelite FLC assays were calculated using the outcomes of the IFE analysis. The clinical sensitivity, specificity, and percent agreement were 72.2%, 93.6%, and 82.8%, respectively, for the Freelite assay, and 64.6%, 100%, and 82.2%, respectively, for the N Latex assay, indicating that the Freelite assay has higher clinical sensitivity, whereas the Latex assay has higher specificity, with the percent agreement being almost comparable (Table 3).

In the IFE-positive specimens, 20 cases with a normal FLC ratio in both assays were investigated. Six cases showed extremely low concentrations for the M-band on IFE, and 4 cases manifested intact immunoglobulin multiple myeloma (IIMM) with bound heavy and light chains. Among 5 cases of the IgM-type MG, the FLC ratios were in normal ranges in 4 cases. In addition, 3 cases of lambda-type MG, concomitant with diseases such as CKD or SLE, showed either normal or even increased ratios. Lastly, in the case of the biclonal-type MG that involves both kappa and lambda, the *κ*/*λ* ratio shifted to the M-type in higher concentration.

## 4. Discussion

We compared two FLC assays that have been utilized in diagnosing and monitoring plasma cell dyscrasia. The study populations consisted entirely of patients with or without monoclonal gammopathy and no normal healthy individuals were included. This is particularly important to calculate the specificity of the laboratory tests in clinical situations and our results could give more helpful information in the interpretations of FLC assays in patients with various diseases.

The results of the Freelite assay were distributed over 3–10 times wider than those of the Latex assay. This finding may be explained by the differences in the specificities and affinity owing to the different antibody specificity for monoclonal and polyclonal reagents [10]. Because of these considerable differences in the range of the results, these FLC assays could not be switched with each other during monitoring patients [11]. We have designed the method of this study to perform the next dilution step in addition to the autodilution steps to avoid the antigen excess problems. As a result, we could not experience any problems related to antigen excess in all study cases and the data could be analyzed entirely on the aspects of clinical utility.

In this study, the kappa FLC showed the best correlation between two assays for all specimens, followed by the *κ*/*λ* ratio and the lambda. On comparing the FLC levels according to the group, the kappa FLC showed high correlation in both groups, with the correlation coefficient being greater than 0.95, and the lambda FLC of the MG group also showed the correlation coefficient, 0.8. The *κ*/*λ* ratio of the non-MG group showed the lowest correlation, *r* = 0.52, and this is in line with the international guidelines, according to which changes in the ratio in the normal ranges should not be considered as clinically significant [3, 4]. In a previous study that compared the two assays, correlation coefficients were around 0.9 for kappa and 0.7 for lambda and *κ*/*λ* ratio, indicating that the current study had relatively better outcomes [9]. This can be explained by the fact that the present study had a higher proportion of MG group specimen than the previous study.

In the concordance analysis, the overall concordance rate of the *κ*, *λ*, and *κ*/*λ* ratio between the two assays was approximately 70–80%, which was lower than one previous study, approximately 90% [9]. This result is also believed to be associated with the proportion of the groups. The non-MG group showed higher concordance rates in all the *κ*, *λ*, and *κ*/*λ* ratios compared to those of the MG group. Four cases in the non-MG group showed a discordant *κ*/*λ* ratio and were shown to be false positives in the Freelite assay. This would be relevant to broader ranges of the Freelite assay outcomes compared to those of the N Latex assay. Among the 10 discordant cases for the lambda assay in the MG group, 8 cases had very high kappa concentration in the N Latex assay. These lambda results presented false positives, indicating the same patterns reported in a previous study [6].

Comparisons of the *κ*/*λ* ratio and IFE results showed that the Freelite assay had higher clinical sensitivity than the N Latex assay, that is, 72.2% versus 64.6%, and the N Latex assay showed 100% specificity, compared to 93.6% for the Freelite assay. These results were similar with the results of a previous study [11]. The agreement rate with the IFE results was comparable in both assays, approximately 82%. The cases in disagreement with the IFE results were found in those of very low concentration of the M-proteins, IIMM, MG with CKD or polyclonal gammopathy, biclonal MG, and most cases of the IgM-type MG. Some of these findings, CKD or polyclonal gammopathy and IgM-type MG, were reported in previous studies [11, 12]. This study has extended our knowledge about the clinical settings which limit the usefulness of the FLC assays in detecting MG. Therefore, it is essential to perform the IFE along with FLC assay for detecting M-protein in such cases as very low levels of the M-protein, CKD, polyclonal gammopathy, biclonal MG, and IgM-type MG.

In terms of the limitations of this study, it may be pointed out that the MG diseases with low incidence rates such as nonsecretory myeloma, amyloidosis, and solitary plasmacytoma could not be sufficiently included on account of the relatively small sample size. By conducting large-scale studies, clinical implications of the FLC measurement for the disease group with low incidence rate will have to be further clarified.

In conclusion, the N Latex assay and the Freelite assay showed good correlations and concordance rates. When the *κ*/*λ* ratios of FLC assays and the IFE were compared, the Freelite assay showed higher clinical sensitivity and the N Latex assay showed higher specificity. Because of the obvious differences in the dynamic range between the assays, the same kind of assay should be employed for monitoring MG patients during follow-up period. Lastly, both FLC assays seem to have limited clinical utility in detecting MG in certain clinical settings.

## Figures and Tables

**Table 1:** Study group of 120 patients.

Group	Diagnosis	Number of patients
Monoclonal gammopathy (MG)	Multiple myeloma	35
	Light chain myeloma	10
	MGUS	9
	Non-Hodgkin's lymphoma	4
	Amyloidosis	2
	Plasmacytoma	2
	Waldenström's macroglobulinemia	1
	Total	**63**

No monoclonal gammopathy (non-MG)	Chronic kidney disease	10
	Bone fracture	8
	Pneumonia	7
	Cancer	4
	Neuropathy	3
	Cerebral hemorrhage	3
	Anemia of chronic disease	2
	Iron deficiency anemia	2
	Autoimmune diseases	2
	Parkinson's diseases	2
	Miscellaneous	13
	Total	**57**

MGUS: monoclonal gammopathy of unknown significance.

**Table 2:** Method comparison between the N Latex FLC assays and the Freelite assays.

Group	*N*	N Latex FLC, mg/L	Freelite FLC, mg/L	Pearson's correlation (*r*)	Passing-Bablok slope (95% CI)	Normalized difference (%)
All						
FLC *κ*	157	2.3–8920.0	6.0–29200.0	0.9792	0.76 (0.71–0.80)	−4.6
FLC *λ*	157	2.6–599.0	1.0–2590.0	0.8264	1.18 (0.99–1.35)	5.3
*κ*/*λ* ratio	157	0.004–323.2	0.008–4687.0	0.9064	0.45 (0.37–0.51)	−10.1
Monoclonal gammopathy						
FLC *κ*	100	2.3–8920.0	6.0–29200.0	0.9793	0.67 (0.61–0.75)	−5.2
FLC *λ*	100	2.6–599.0	1.0–2590.0	0.8388	0.78 (0.69–0.92)	0.43
*κ*/*λ* ratio	100	0.004–323.2	0.008–4687.0	0.9065	0.43 (0.35–0.53)	−8.0
No monoclonal gammopathy						
FLC *κ*	57	7.2–222.0	9.5–269.0	0.9867	0.78 (0.73–0.82)	−4.0
FLC *λ*	57	11.4–358.0	13.9–213.0	0.9286	1.72 (1.53–1.94)	8.15
*κ*/*λ* ratio	57	0.261–1.243	0.646–4.347	0.5168	0.47 (0.28–0.71)	−11.2

**Table 3:** Clinical sensitivity and specificity between FLC assay and immunofixation electrophoresis in 157 sera.

N Latex FLC assay	IFE			Freelite assay	IFE		
	Positive	Negative	Total		Positive	Negative	Total
Abnormal	51	0	51	Abnormal	57	5	62
Normal	28	78	106	Normal	22	73	95
Total	**79**	**78**	**157**	Total	**79**	**78**	**157**

N Latex FLC assay: agreement, 82.2%; sensitivity, 64.6%; specificity, 100%. Freelite assay: agreement, 82.8%; sensitivity, 72.2%; specificity, 93.6%.

IFE: immunofixation electrophoresis.
